# 3D Visualization as a Communicative Aid in Pharmaceutical Advice-Giving over Distance

**DOI:** 10.2196/jmir.1437

**Published:** 2011-07-18

**Authors:** Martin Östlund, Nils Dahlbäck, Göran Ingemar Petersson

**Affiliations:** ^1^eHealth InstituteLinnaeus UniversityKalmarSweden; ^2^School of Computer Science, Physics and MathematicsLinnaeus UniversityKalmarSweden; ^3^Department of Computer and Information ScienceLinköping UniversityLinköpingSweden

**Keywords:** Pharmaceutical instruction, 3D visualization, distance communication

## Abstract

**Background:**

Medication misuse results in considerable problems for both patient and society. It is a complex problem with many contributing factors, including timely access to product information.

**Objective:**

To investigate the value of 3-dimensional (3D) visualization paired with video conferencing as a tool for pharmaceutical advice over distance in terms of accessibility and ease of use for the advice seeker.

**Methods:**

We created a Web-based communication service called AssistancePlus that allows an advisor to demonstrate the physical handling of a complex pharmaceutical product to an advice seeker with the aid of 3D visualization and audio/video conferencing. AssistancePlus was tested in 2 separate user studies performed in a usability lab, under realistic settings and emulating a real usage situation. In the first study, 10 pharmacy students were assisted by 2 advisors from the Swedish National Co-operation of Pharmacies’ call centre on the use of an asthma inhaler. The student-advisor interview sessions were filmed on video to qualitatively explore their experience of giving and receiving advice with the aid of 3D visualization. In the second study, 3 advisors from the same call centre instructed 23 participants recruited from the general public on the use of 2 products: (1) an insulin injection pen, and (2) a growth hormone injection syringe. First, participants received advice on one product in an audio-recorded telephone call and for the other product in a video-recorded AssistancePlus session (product order balanced). In conjunction with the AssistancePlus session, participants answered a questionnaire regarding accessibility, perceived expressiveness, and general usefulness of 3D visualization for advice-giving over distance compared with the telephone and were given a short interview focusing on their experience of the 3D features.

**Results:**

In both studies, participants found the AssistancePlus service helpful in providing clear and exact instructions. In the second study, directly comparing AssistancePlus and the telephone, AssistancePlus was judged positively for ease of communication (*P* = .001), personal contact (*P* = .001), explanatory power (*P* < .001), and efficiency (*P* < .001). Participants in both studies said that they would welcome this type of service as an alternative to the telephone and to face-to-face interaction when a physical meeting is not possible or not convenient. However, although AssistancePlus was considered as easy to use as the telephone, they would choose AssistancePlus over the telephone only when the complexity of the question demanded the higher level of expressiveness it offers. For simpler questions, a simpler service was preferred.

**Conclusions:**

3D visualization paired with video conferencing can be useful for advice-giving over distance, specifically for issues that require a higher level of communicative expressiveness than the telephone can offer. 3D-supported advice-giving can increase the range of issues that can be handled over distance and thus improve access to product information.

## Introduction

People are not very good at using their medications correctly. It has been shown that only about 50% of all patients manage to follow the instructions they have been given to the full [1-3]. The effects of this failure to comply with given instructions are serious. Nearly half of all medication-related admissions to hospital are directly related to the patients not following their prescriptions [4]; and 8%–10% of the total number of hospital admissions are directly related to incorrect use of medication [5]. Many different strategies have been attempted to improve on the situation. In a comprehensive review of intervention studies, Haynes et al [6] list the following interventions as the most promising to improve compliance: providing more and better instructions, fostering a good relationship between the health care professional and the patient, scheduling regular follow-up meetings, and supplying the patient with appropriate reminders. The list shows that patient–caregiver communication is very important in fostering good use of medication.

Many types of communication tools have been tried out in the health care domain, and experience shows that the technology does not need to be advanced to be helpful. Indeed, many positive results have been achieved with simple solutions. For instance, many studies have investigated the usefulness of email-type messaging between patient and physician [7-11] and, of course, the telephone is a well-established tool for patient–caregiver consultation [12,13]. More sophisticated communication technologies such as video conferencing and groupware solutions have also been used, for example to review patient data [14,15] or to discuss medical imaging data such as from scans [16]. Results have been positive and, although initial costs can be high for the more high-tech variants, distance communication solutions of this kind have generally been shown to be cost effective [17-19].

In this paper we explore the usefulness of 3-dimensional (3D) visualization technology as a communication tool. 3D technology has been put to good use in medicine, for example in the exploration of scanning data [16], laparoscopic surgery training [20], and phobia therapy [21]. However, it has not been used to any great extent as an instrument of communication. In other domains, most prominently that of computer-aided design [22], it has been used as a communication enhancer for some time, for example to support remote collaboration between geographically dispersed teams [23-25], to review designs over distance [26-28], and to offer training and support to remotely located service personnel [29].

One exception where 3D technology has been used to support communication in health care is with the use of 3D virtual worlds such as Second Life. Second Life has been used in medical training to provide a virtual meeting place for health-related dialogue and for disseminating health care information [30,31]. This is an interesting exploration into the realm of possibilities that 3D technologies offer, but it is not a practical solution for most types of caregiver–patient communication.

In the health care domain, the preference seems to have been either for technical solutions that are very simple, such as email-based messaging systems, or for those that are very advanced, such as state-of-the-art video conferencing systems or virtual worlds. The problem is that *simple* solutions might reach many people, but the expressive power is often limited. *Advanced* solutions have more expressive power, but the number of people who can be reached may be limited because they might not have the required hardware or software, or they find it difficult to use. The challenge is to find a solution that gives room for expressiveness but also can be made available for a broad audience.

The objective of the present exploratory descriptive study was to examine the value of 3D visualization as a communicative aid in pharmaceutical advice-giving over distance. We created a Web-based application named AssistancePlus that uses interactive 3D representations to demonstrate handling instructions for complex pharmaceutical products. For the application to be useful in practical terms, it needs to be easy to access and easy to use for members of the general public. Thus, the application also presents a proof-of-concept that an advanced technology such as 3D visualization can be packaged and presented in such a way that the resulting application is accessible to this group. We carried out two evaluation studies to investigate its practical usefulness for pharmaceutical advice-giving. Comparisons were made with telephone communication and face-to-face interaction in terms of expressiveness and accessibility.

## Methods

### Materials

AssistancePlus is a communication tool that uses co-manipulation of 3D representations of products, audio/video communication, and co-browsing to support advice-giving on pharmaceutical products over distance. Building on Clark’s theory of communication and specifically his theory of common ground [32-34], we believe that using interactive virtual 3D objects together with audio/video communication will support communicative efficiency by creating a sense of *co-presence* between the patient and advisor. Our hope is that this will translate into a natural feel to the communication, as well as providing a high level of expressiveness. 

AssistancePlus has been tailored to meet the specific needs of the 2 parties involved in the advice-giving interchange: the *advice seeker* and the *advisor.* The advice seeker is typically a novice and a beginner—a novice to the content domain and a beginner at using the communication tools at hand. The advisor is an expert and a professional*—*an expert in the content domain and a professional at using the communication tools (see [35,36] for a similar description of the advice seeker–advisor relationship). Due to the different nature of the 2 roles, their needs and wants are different. The advice seeker wants a service that is easy to access and easy to use. The advisor wants a service that allows him or her to communicate efficiently and effectively. This is achieved easily enough by providing them with different versions of the user interface. However, although it is true that most of the information in an advice-giving setting flows from the person giving advice to the person receiving advice, it is also necessary to have a flow in the other direction. No advice-giving session should be considered complete until the advice seeker has *internalized* the given information—that is, not only has received the information, but also has understood it and is motivated to act in accordance with what has been learned (see, for example, Clark [32,33]). It must be the responsibility of the advisor in the role of expert professional to make sure that this is so, and to do this the advisor needs feedback from the advice seeker. Therefore, it is important that advice seekers also can be expressive so that they can signal or demonstrate their understanding back to the advisor. The challenge here is to provide the right level of expressiveness for each party and, for the advice seeker, to find an appropriate balance between ease of use and expressive power. Details on how this challenge was approached in the design of AssistancePlus can be found in Multimedia Appendix 1, together with a technical description of the service.

**Figure 1:**
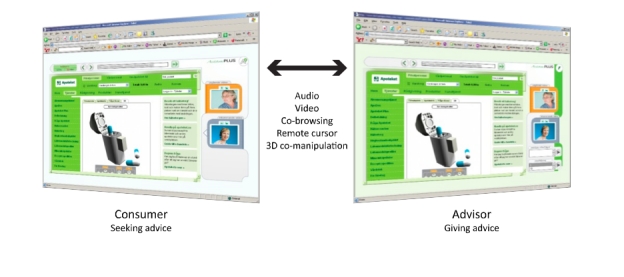
AssistancePlus, a tool for 3-dimensional (3D) -supported advice-giving on pharmaceutical products over distance

Figure 1 shows a screenshot of AssistancePlus with the first version of the 3D player being used to demonstrate handling instructions for an asthma inhaler. The main features of AssistancePlus are (1) co-manipulation of shared 3D objects representing pharmaceutical products, (2) two-way text and audio/video communication, and (3) co-browsing of webpages with synchronized page scrolling and remote cursors

### Evaluation Studies

The value of 3D-supported advice-giving was assessed in 2 separate studies, both set up in the same way. Study participants (advice seekers) were exposed to AssistancePlus in a realistic usage situation after which they were questioned about their experience. The first study was explorative and probed the general usefulness of 3D visualization for advice-giving purposes. The second study was focused on the details of how 3D visualization contributed to raising the level of expressive power. A telephone session was included in the second study to provide an explicit (and controlled) reference point with which to compare their experience with AssistancePlus. The type of questions that AssistancePlus is appropriate for was also examined. A large amount of data was collected in the 2 studies, of which a selection is presented here (the full range of the data is presented in [37]).

### User Study I: Simple 3D Player

The objective of this substudy was to broadly explore the issue of how 3D visualization can be used as a tool for advice-giving on pharmaceutical products.

#### Materials

**Figure 2:**
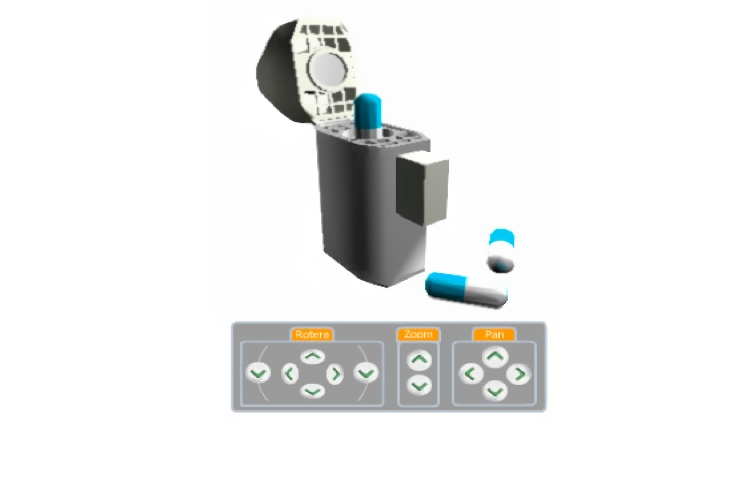
The Ingelheim asthma inhaler displayed using the first version of the 3-dimensional player

The AssistancePlus application, with version 1 of the 3D player, allowed advice seekers to communicate with an advisor over distance using 2-way audio/video communication and co-browsing of webpages (see Multimedia Appendix 1 for a description of AssistancePlus and the 3D player). We made 2 webpages available for the advice seekers and adviser to browse together: (1) a text-based page with general information about the product (side effects, storage directions, etc), and (2) a page with a 3D model of an asthma inhaler (Ingelheim; Boehringer Ingelheim GmbH, Ingelheim, Germany) (Figure 2).

#### Participants and Recruitment

Playing the role of advice seeker were a group of 10 pharmacy students (9 women and 1 man; age range 22–40 years). Pharmacy students, with obvious preknowledge of the subject area, were selected because their professional insight into the subject area was considered helpful at this explorative stage (the pharmacy students had no connection with the AssistancePlus project and received no reward for participating in the study). The students assumed the role of a relative of an asthma patient (the role of relative is relevant because many people are involved in their relations’ medication, and it was a more realistic role for the pharmacy students to play than patient). The role of advisor was assumed by 2 licensed pharmacists from the National Cooperation of Swedish Pharmacies’ call centre (both women; age range 40–50 years, each with several years’ experience with telephone consultation).

#### Procedure

The study was performed in a usability lab using standard computer equipment and software (Web browser). Advice seekers and advisors were in separate rooms and did not meet each other before or after the trial. Advice seekers, who were assigned the task of trying to find out as much as possible about the inhaler, interacted through AssistancePlus for 15 minutes with a randomly assigned advisor. No special instructions were given on how much time to spend on any specific topic. The first author conducted individual in-depth, semistructured interviews on a separate occasion, 1 hour each for the pharmacy students and 2 hours each for the advisors.

#### Data Collection

The students and advisors were filmed on video to allow for analysis of the advice seeker–advisor interaction. The video on the students’ side recorded keyboard and mouse use as well as the screen; and the video on the advisor side recorded only the screen. The audio from the interview was recorded, transcribed, and summarized.

A semistructured interview was administered on a separate occasion. The interview covered the following areas: general impressions; specific impressions concerning audio and video features, co-browsing features, and 3D; locus of control (self/advisor); level of activeness (self/advisor); usefulness of service; and target audience.

### User Study II: Advanced 3D Player and Comparison With Telephone

The objective of this substudy was to compare the experience of using AssistancePlus with that of the telephone; to identify the type of questions for which use of AssistancePlus is appropriate; and to investigate the dynamics of how 3D visualization contributes to communicative expressiveness.

#### Materials

**Figure 3:**
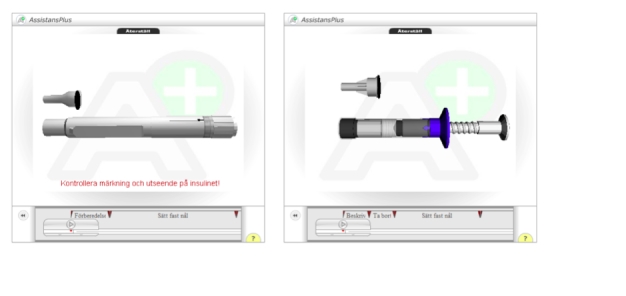
The 2 pharmaceutical products used in the second user study displayed using the second version of the 3-dimensional player: Lantus OptiSet (left) and Genotropin MiniQuick (right)

As in the first study, AssistancePlus, with the second version of the 3D player, was used to allow advice seekers to communicate with an advisor over distance with 2-way audio/video communication and co-browsing (Multimedia Appendix 1). As in the first study, 2 webpages were available to the participants: (1) a text-based page with general information (side effects, storage directions, etc), and (2) a page with a 3D model, this time either of a pen injector for insulin glargine (Lantus OptiSet, sanofi-aventis, Paris, France) or of a disposable syringe for human growth hormone equivalent (Genotropin MiniQuick, Pfizer, New York, USA). Figure 3 shows screenshots of their respective 3D representations as displayed with the second version of the 3D player (Multimedia Appendix 1; see also Multimedia Appendix 1 for additional screenshots of the 3D sequences used).

#### Participants and Recruitment

For the role of advice seeker, 23 participants were recruited from various night-school classes: 13 women and 10 men with varied backgrounds, interests, and ages (18–74) (participants received a nominal gift in the form of a lottery ticket with a value of less than $10). As in the first study, the participants played the role of a relative, this time of a patient with either diabetes (Lantus OptiSet) or a growth hormone deficit (Genotropin MiniQuick). The role of advisor was assumed by 3 licensed pharmacists from the National Cooperation of Swedish Pharmacies’ call centre (2 women, ages 36 and 40 years; and 1 man, age 50 years), each with several years’ experience with telephone consultation.

#### Procedure

The advice seekers were instructed to try to find out as much as possible about the 2 products, first using the telephone for one product and then, on a separate occasion, using AssistancePlus for the other product. The telephone condition was first for all participants. The telephone session was conducted from home, while the AssistancePlus session was carried out in a usability lab. Advice seekers and advisors were in separate rooms and did not meet each other before or after the trial. Each session lasted 10 minutes. The order of the products was balanced, with 12 advice seekers starting with Lantus OptiSet and 11 starting with Genotropin MiniQuick. The advisors were randomly assigned with a different advisor in the AssistancePlus session and the telephone session. After the AssistancePlus session, the advice seekers filled in a paper-based questionnaire and then participated in a 20-minute semistructured interview led by the first author.

#### Data Collection

The audio from the telephone sessions was recorded. The AssistancePlus sessions were filmed on video and the screen image was recorded. Questionnaire data were collected and the audio from the interviews was recorded. The questionnaire covered the following areas: background demographic variables, general attitudes toward computers and the Web, impressions of AssistancePlus and its 3D features, comparisons between the telephone and the AssistancePlus session, and which channel (medium) they would use to inform themselves about pharmaceutical products of varying complexity. In the comparison between the telephone and the AssistancePlus session, participants were asked to distribute 10 points between the telephone and AssistancePlus for each of the following 7 factors: ease of use, ease of communication, sense of personal contact, explanatory power, level of understanding, level of trust, and efficiency. For example, if AssistancePlus is given a rating of 6 on ease of use, the rating for the telephone for this factor must be 4, to make a total of 10. (The factors were gleaned from a discussion seminar held with representatives from the pharmaceutical industry, health care representatives, and advice-giving professionals [38].) Participants were also asked which channel they would choose to inform themselves about products of varying complexity level. In addition to telephone and AssistancePlus, they were able to choose from pharmacy store and the Internet, which were added to provide a more complete range of alternatives (note that the participants’ acquaintance with AssistancePlus and telephone channels was controlled in the study, but that with the Internet and the pharmacy store channels were based on uncontrolled personal experience). Participants were asked to distribute 10 points among these 4 channels for each of the following 4 product types: simple nonprescription medications, simple prescription medications, complex prescription medications, and complex medications requiring handling. The interview questions focused on their experience of the 3D features and covered the following areas: perceived level of expressiveness, experience of 3D content, sense of presence, level of activeness, and locus of control.

### Statistical Analysis

For each of the 7 factors on which telephone and AssistancePlus were compared, a set of preference scores were calculated by subtracting the rating given to the telephone from that given to AssistancePlus (a positive preference score thus indicating a preference for AssistancePlus and a negative score indicating a preference for the telephone). Wilcoxon signed rank test was used to assess the statistical significance of the preference scores. Friedman tests were used to assess whether the complexity level of the product affected the choice of communication channel. Descriptive statistics were calculated for other survey data.

## Results

### Findings From User Study I: Simple 3D Player

Being accustomed to telephone consultation, the 2 advisors could make an experience-based comparison between using the telephone and using AssistancePlus to give advice. The advisors welcomed the power of expression the 3D content provided. They felt that this extra expressiveness made it possible to handle more complex issues than could be dealt with using the telephone. In particular, the advisors valued the potential for increased precision, for example that they could rotate and zoom in on the model and then use their remote cursor to point to the exact detail they were referring to in their spoken communication.

The participants in the role of advice seekers were also pleased with their experience of AssistancePlus, both in general and with the 3D features specifically. All of the participants found the 3D features useful as a pedagogical aid and found the experience informative. The reasons they gave varied in wording, but centered on issues of clearness and distinctiveness. Several of the participants (7/10) also described the interaction as similar in feel to that of a face-to-face conversation concerning both level of expressiveness and sense of personal contact.

One somewhat surprising finding was that the activity level of the advice seekers was seemingly not very high. None of them attempted to use the 3D controls themselves and only 1 used the remote cursor purposefully. Reasons for not being more active given in the interviews were that they felt they might disrupt the advisor (6/10 participants), that the content “belonged” to the advisor (5/10), and that they felt a bit intimidated by the many buttons on the 3D player control panel (3/10). However, the main reason was that they did not feel it necessary to be more active than they were. They were active with their voice and, if they wanted to see a different view of the 3D model, they could simply ask the advisor. This said, the advice seekers wanted to keep the feature (9/10). It seems that knowing that they had the option to control the model made them feel more involved in the advice-giving process (even if they did not actually do this). Similarly, the advisors felt that, while the vocal feedback the advice seekers gave was sufficient for the advisors to judge understanding in the present situation, in other situations it might be helpful having the advice seekers control the model themselves to demonstrate what they have learned.

### Findings From User Study II: Advanced 3D Player and Comparison With Telephone

**Figure 4:**
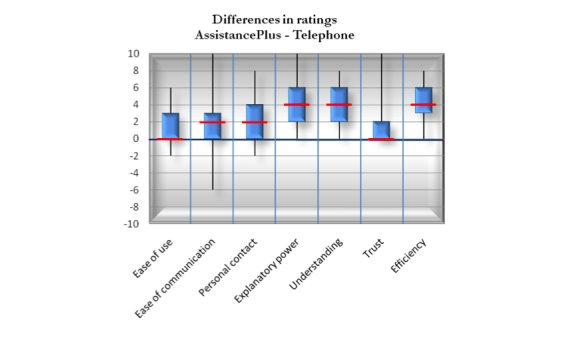
Preference scores comparing AssistancePlus and telephone. Positive scores (above the 0 baseline) indicate that AssistancePlus received a higher rating. The vertical lines show the total range, the filled blue sections show the interquartile ranges, and the horizontal red lines show the median values

Figure 4 shows the median value, interquartile range, and total range of the preference scores that were calculated by subtracting telephone ratings from AssistancePlus ratings for each of the 7 factors on which telephone and AssistancePlus were compared (see Methods section). All the preference scores were positive in AssistancePlus’ favor, and all scores except *e* *ase of use* were shown to be statistically significant (Table 1). For *ease of communication*, *personal contact,* and *trust*, the magnitude was moderate; for *explanatory power*, *understanding,* and *efficiency* it was more pronounced. The results were corroborated in the interviews, where participants described their experience with AssistancePlus as being more clear and expressive, being more personal and present, and giving a deeper sense of understanding. Also, several participants described the feel of the interaction when using AssistancePlus as being more similar to a face-to-face meeting than that of a telephone conversation. It should be noted, though, that both the questionnaire and the interview were administered in conjunction with the AssistancePlus session, which might have introduced a positive bias in AssistancePlus’ favor, being the more recently experienced.

**Table 1:** Preference scores comparing AssistancePlus and telephone (N = 23)

Factor	Median (quartile deviation)	Mean (SD)	Z score (Wilcoxon signed-rank test)^a^	2-tailed^b^ *P* value
Ease of use	0 (3)	1.04 (2.69)	–1.596	.110
Ease of communication	2 (3)	2.26 (2.58)	–3.448	.001
Personal contact	2 (4)	2.26 (2.43)	–3.454	.001
Explanatory power	4 (4)	4. 22 (2.63)	–4.042	< .001
Understanding	4 (4)	3.57 (2.63)	–3.758	< .001
Trust	0 (2)	1.48 (2.78)	–2.401	.016
Efficiency	4 (3)	3.96 (2.06)	–4.078	< .001

^a^Based on negative ranks for [AssistancePlus score] – [telephone score].

^b^H_0_: no difference between scores.

**Figure 5:**
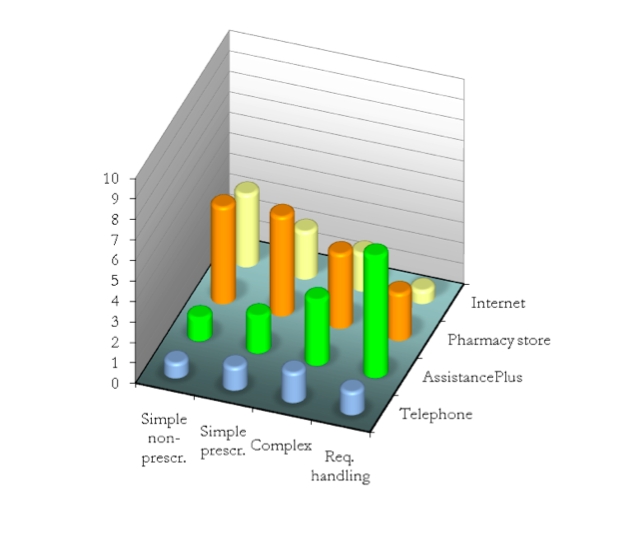
Relative preferences among information channels for different levels of product complexity (prescr = prescription, req = requiring)

Figure 5 displays the results of the preference ratings given for the 4 types of products and 4 different channels. On type of product (front to back in Figure 5), the variation was found to be significant for all product categories except *c* *omplex medications* (Table 2). On type of channel (left to right in Figure 5), *AssistancePlus* and *Internet* differed significantly, but *t* *elephone* and *p* *harmacy store* did not*.* The results indicate that the advice seekers prefer a simple channel for simpler questions and a more powerful channel for more complex questions—that is, that they make their choice with consideration to the complexity of the issue at hand.

Somewhat surprisingly, though, the advice seekers did *not* rate the face-to-face setting—ostensibly the most expressive channel among the alternatives—as the preferred channel for the most complex type. In fact, the results instead showed an inverse relationship between the *pharmacy store* and product complexity. The reason for this was revealed in the interviews. The participants saw the meeting in the pharmacy store not as an expressive and personal face-to-face interaction, but as a fleeting encounter in a nonprivate, noisy, and stressful environment. The telephone received surprisingly low ratings for all categories indicating that the telephone is simply not a popular alternative when seeking medical advice.

**Table 2:** Friedman tests on preferences among information channels (N = 23)

Type of product		Telephone	AssistancePlus	Pharmacy store	Internet	Friedman test (c^2^)^a^	*P* value
Simple nonprescription	Mean	0.70	1.09	4.70	3.52	22.35	< .001
	SD	1.06	1.20	3.42	3.25		
Simple prescription	Mean	1.09	1.78	4.83	2.30	17.15	.001
	SD	1.35	1.93	2.95	2.40		
Complex	Mean	1.43	3.17	3.61	1.83	7.608	.06
	SD	1.56	2.98	2.66	1.83		
Requiring handling	Mean	1.09	5.91	2.27	0.61	43.05	< .001
	SD	1.31	2.25	1.86	0.89		
Friedman test (c^2^)^a^		3.329	43.01	5.949	24.53		
	*P* value	.36	.001	.11	.001		

^a^ Two-tailed; df = 3 for all tests.

The level of activity on the part of the advice seekers was very low in the first study, with none of them even attempting to use the 3D controls. This behavior changed in the second study, where they were more active. Typically, the actions they performed were simple ones such as using their remote cursor to point to details on the 3D model (16/23, 70% of participants), moving the playhead to go to a specific point in the animation (2/23, 9%), and starting and stopping playback (3/23, 13%). Only 1 participant used the click-and-drag controls to rotate and zoom in on the model. However, the preference for simpler action was not because they did not know how to perform these more advanced actions. As in the first study, they simply found it easier to have the advisor do it for them. Still, the participants were very positive about having the option to be able to control the 3D content themselves (N = 23, mean [SD] 5.42 [0.90], range 1–6) and wanted to keep the feature. The advisors were also pleased that the advice seekers had the option to control the 3D content themselves.

## Discussion

We have shown that 3D visualization techniques paired with video conferencing can be useful in supporting advice-giving over distance and can be used even in low-bandwidth settings. AssistancePlus offers a proof-of-concept of how 3D visualization can be used to extend the range of questions about pharmaceutical products that can be handled over distance. Distance communication cannot, and should not, be used to replace the physical meeting between patient and caregiver, but in many situations distance communication can save time and resources and give patients quicker and easier access to the information they need. Haynes et al [6] listed more and better information and fostering a good relationship with caregivers as the 2 top interventions to improve compliance. We believe that a service such as AssistancePlus can contribute to both of these by making it easier for patients or clients to communicate with their caregivers. We also found it interesting that in some cases our participants actually preferred AssistancePlus to face-to-face communication, as this usually takes place at the pharmacy. This illustrates our belief that Internet-based communication not only should be seen as a substitute for face-to-face interaction but also can in some cases offer a better alternative.

### Choice of Channel Based on Question Content

Advice seekers and advisors alike found AssistancePlus useful as a pedagogical aid for advice-giving and particularly appreciated the clarity and exactness brought to instructions by the 3D features. This does not mean that AssistancePlus is the right choice for all types of advice-giving situations. The results from the second study indicate that information seekers do not just go for the most expressive channel, but differentiate their choice. Perceived level of expressiveness is one factor affecting choice, but other factors are also important. For instance, the pharmacy store was an unpopular alternative for the reason that it was seen as lacking in privacy. If we try to isolate the issue of level of expressiveness here, it seems that participants preferred the channel that offers not too much, not too little, but just the right level of expressiveness. The implication is that we should provide advice seekers not with a single catch-all channel, but with a selection so that they can choose the right channel for the question at hand. Of course, the downside of providing choice is that it places a burden on the advice seeker to choose among an ever-increasing range of channels. We believe that a major future challenge will be to provide guidance services to help advice and information seekers to find not only the right information, but also the right channel.

### Empowering the Advice Seeker

In the advice-giving situation, most of the communication naturally flows from the person giving the advice to the person receiving the advice, but we have argued that it is important that the advice seeker provide feedback so that the advisor can properly judge understanding and motivation. Here, care must be taken not to overwhelm or intimidate the advice seeker, who perhaps for the first time is communicating over distance using advanced technology. Our solution is to provide self-use of, for example, the 3D controls as an option, something that one can do, but does not have to do.

An interesting—and we believe important—finding in this area was that advice seekers did feel more involved when presented with the option of using the 3D controls, but this sense of involvement was not the result of actual action. Instead it seemed to stem from the awareness that they could be active. Both those who actively used the 3D controls and those who did not felt empowered by knowing they could control the 3D content (theoretical support for this empowerment effect can be found in Clark’s theory of common ground [32-34]). This finding suggests that it is positive to present advice seekers with more functionality than they are expected to use. However, we hypothesize that the empowerment effect will occur only if the functionality is perceived as something they can manage. This means that either the functionality needs to be simple enough to be self-explanatory, or it can be easily explained by way of demonstration, which is the case with the playback and 3D controls used in the studies. We believe that this finding, provided that it can be corroborated in further studies, can have important consequences for the design of online communication services of the kind presented here.

### Adapting to the Specific Communication Situation

In this paper we have exemplified how 3D visualization can be used in health communication for a specific advice-giving situation. It is in the custom tailoring to the specific communication setting and to its actors that the service gains its edge, but we believe that the concept it presents—ease of access with low technical and usability thresholds and a level of expressiveness tailored to the demands of the specific communication situation—is relevant for any area of health care where there is a need for improving access to information and for bringing patients and caregivers closer to each other.

### Limitation of Study and Cost of Service

It must be noted that both presented studies used convenience samples and were performed in usability laboratories. There may also be a novelty effect, and it remains to be seen how a service such as AssistancePlus would work in a real-world setting with real patients and caregivers who might react differently from the test participants in our studies. That said, the results are promising and we believe AssistancePlus can provide both an alternative to the telephone when it is not expressive enough and a complement to the face-to-face interaction when a physical meeting is not convenient.

We have not performed a detailed analysis of the cost of setting up and running a service such as AssistancePlus, but it is our assessment that it should be comparable with that of setting up a telephone-based call centre. Of course, the easiest way to get a service like AssistancePlus up and running would be to add it to the list of services offered by an existing call centre. Since all the functionality demonstrated in the AssistancePlus application can be implemented using open source free software, this means development and running costs can be kept down and, while the production of the 3D content is an extra cost for each product that is added, it is a cost of moderate size.

### Conclusion

We have shown that 3D visualization techniques paired with video conferencing can be useful in supporting advice-giving over distance and can be used even in low-bandwidth settings. We also showed that 3D visualization brings clarity and exactness to given instructions and contributes to communicative efficiency. It is a technology that can be made available to the general public and, if implemented with proper consideration to the role and character of the advice seeker, will be both easy to access and easy to use and, in some cases, even preferred to face-to-face communication.

## Multimedia Appendix 1

Technical description of AssistancePlus and screenshots of the second version of the 3D player displaying animation sequences for Lantus OptiSet and Genotropin.

## Multimedia Appendix 2

Survey from user study II in English translation (original is in Swedish).

## Abbreviations

3D: 3-dimensional
