# Supplementary material for: 3D Visualization as a Communicative Aid in Pharmaceutical Advice-Giving over Distance
Source: J Med Internet Res. 2011 Jul 18;13(3):e50. doi: 10.2196/jmir.1437 (PMC3222187; doi:10.2196/jmir.1437)

Multimedia Appendix 1

Technical Description of AssistancePlus and the two versions of the 3D player

AssistancePlus is a communication tool for advice-giving over distance about pharmaceutical products. The way it works is that the advice-seeker activates the service by clicking on a link on a web page. This is the only action that the advice-seeker is *required* to perform. All other actions are optional. The advice-seeker is joined by the advisor on the web page and they can surf, or *co-browse*, the web together. Page changes and scrolling are synchronised between their browsers so that they always see the same view of the same web page. They can also see one another’s cursor, which allows them to use their cursors to point to things on the shared web page.

The advice-seeker and advisor can communicate with text, audio, and video. If bandwidth is low, the advisor may use audio only one-way and text communication the other way. This means that AssistancePlus can be used in very low bandwidth situations. The advisor can remotely control audio and video settings (after being granted permission from the advice-seeker), which means that the advice-seeker does not have to worry about any communication settings.

The 3D content is embedded on an ordinary web page using a custom 3D player. The 3D player comes in two versions with different features, but with the same function and purpose: to present a virtual version of a pharmaceutical product that can be used to present and demonstrate function and handling of said product. Both versions require very little bandwidth with small initial download sizes and marginal bandwidth demands for the data exchanged to keep advisor- and advice-seeker versions synchronised. Shockwave technology was used for the 3D content. This does limit the accessibility of the service somewhat. Only about 50% of web users have this plug-in pre-installed (47%, June 2010). However, recent advances in Flash technology make it possible to achieve the same functionality using Flash only, making the service available to practically all web users (99%, June 2010).

3D player, version I

The first version of the 3D player is shown in figure 1 displaying an asthma inhaler. The buttons below the 3D model are used to rotate, pan and zoom. When either party uses the controls, the same action is performed on the other side, thereby synchronising their views of the 3D model. Typically it will be the advisor who controls the model, but the advice-seeker has access to the same controls (and can see how they are used by tracking the advisor’s cursor movements when he/she uses them).

Figure 1. Close-ups of the 3D controls for the first version of the 3D player (displaying an asthma inhaler) and incoming and outgoing video streams.

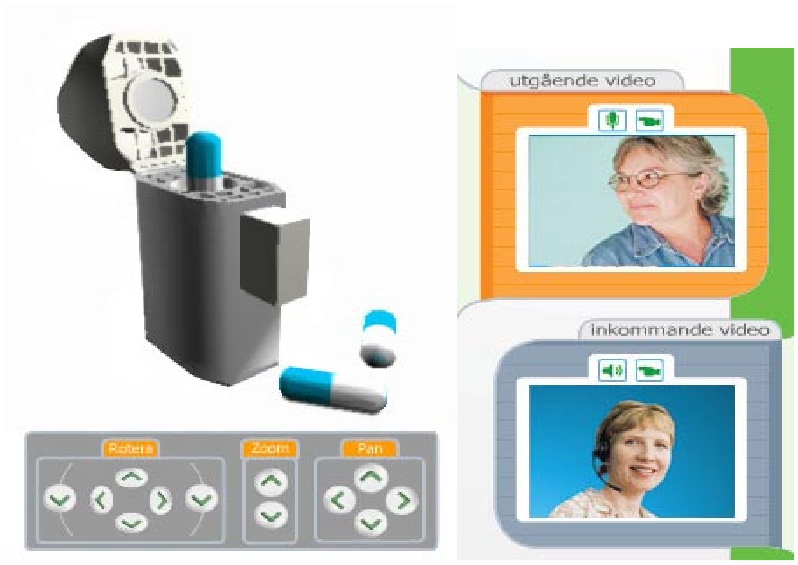

3D player, version II

The second version of the 3D player (see figure 2) uses a click-and-drag system to control rotation, zoom and pan with left-click and horizontal drag for rotation around the y-axis, left-click and vertical drag for zoom, and right-click-and-drag for pan. X- and z-axis rotation was not supported in this version. To make it possible for the advisor to demonstrate the click-and-drag functionality an icon is shown every time the advisor performs a click-and-drag action showing what button was pressed and in which direction the mouse was moved.

3D animation is used to playback series of actions in a video-like sequence. The animation sequences are subdivided into named episodes and are navigated using the timeline below the 3D model, pressing play to start playback of the current episode. Rotation, zoom and pan can be performed independently of the animation playback, i.e. not only when the sequence is paused, but also during playback. Pre-recorded animation was chosen over having a fully interactive model with moveable parts because it allows content to be standardised to a greater degree and it is much easier to control for the user.

The timeline controls are different for the advisor and advice-seeker (see close-ups in figure 3). The interface for the advice-seeker has been kept simple with only play/pause and rewind controls. The playhead can also be dragged to skim through the animation content. The advisor has a more advanced interface with additional controls that add flexibility to the way the animations can be controlled including a drop-down section menu, thumbnail preview images, timeline scrolling and helper notes. The features that are shared by the advice-seeker and the advisor (play/pause and rewind) are in the same position in both interfaces so that the advisor can demonstrate their function by using them.

Figure 2. AssistancePlus with the second version of the 3D player, the advisor’s version to the left and the advice-seeker’s version to the right.

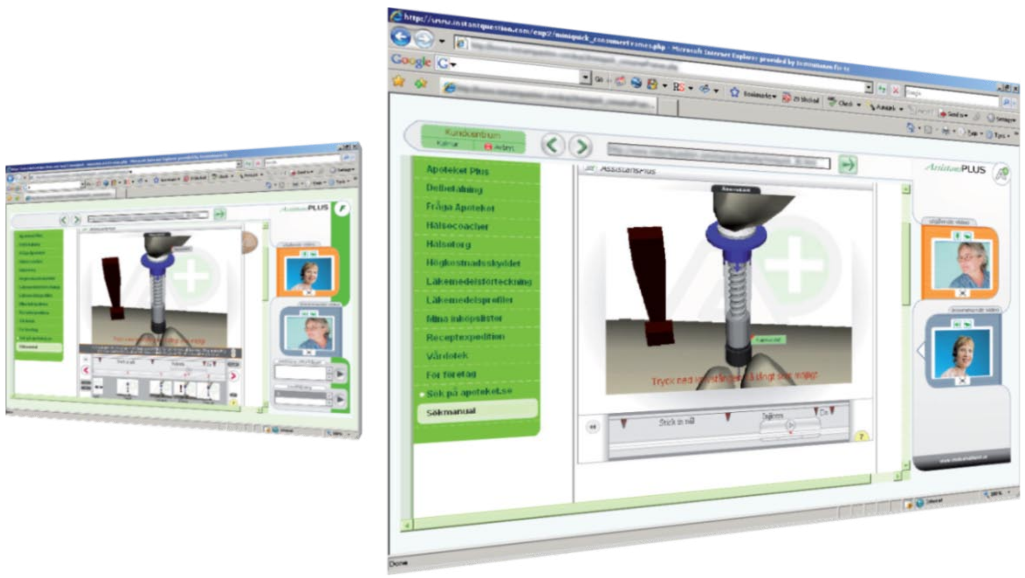

Figure 3. The playback controls for the second version of the 3D player, the advisor's version at the top and the advice-seeker's version at the bottom.

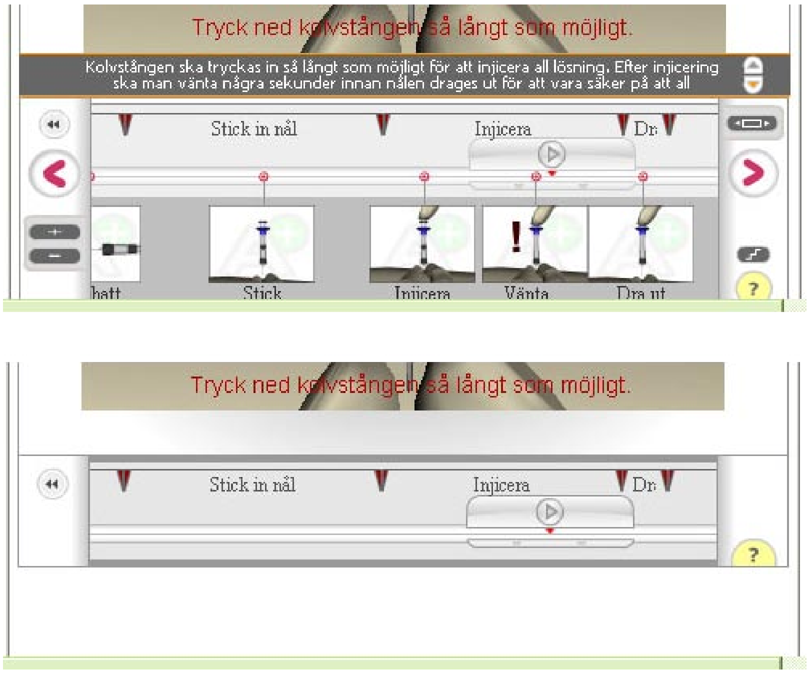

Image sequence for Lantus OptiSet and Genotropin MiniQuick

Lantus OptiSet image sequence:

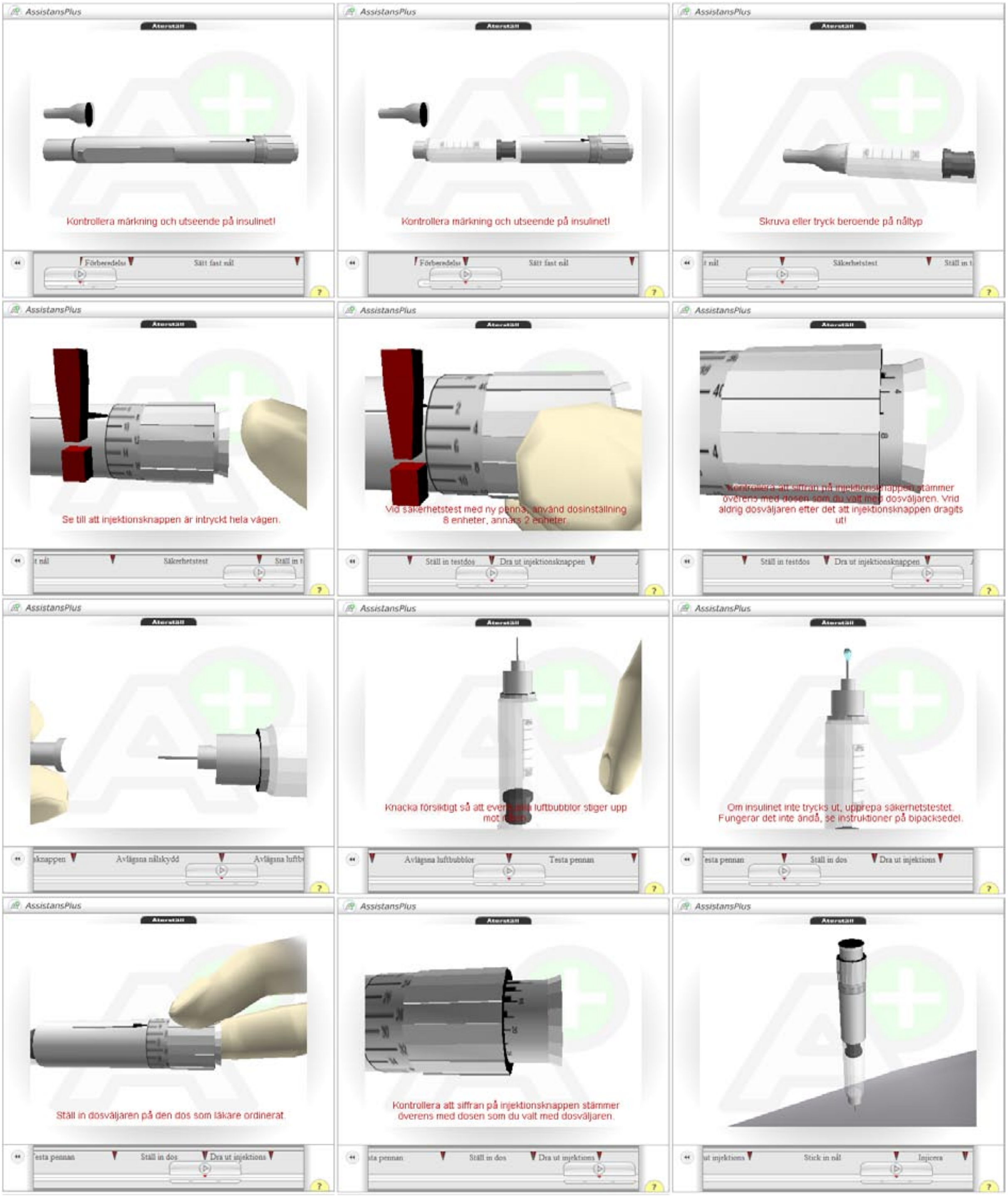

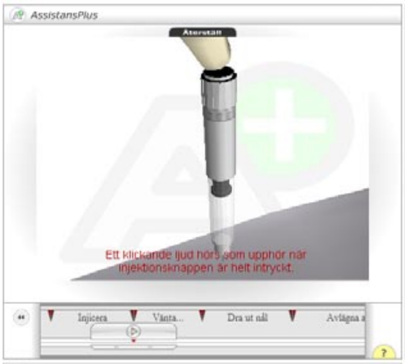
AssistansPlus
Återställ
Ett klickande ljud hörs som upphör när injektionsknappen är helt intryckt.
Injicera
Vänta...
Dra ut nål

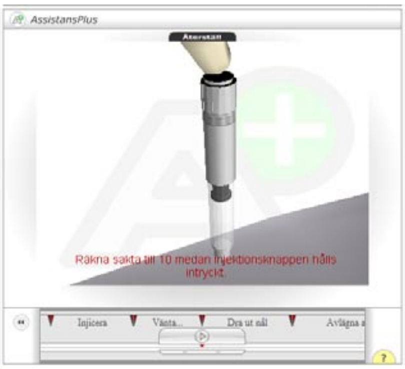
AssistansPlus
Återställ
Räkna sakta till 10 medan injektionsknappen hålls intryckt.
Injicera
Vänta...
Dra ut nål

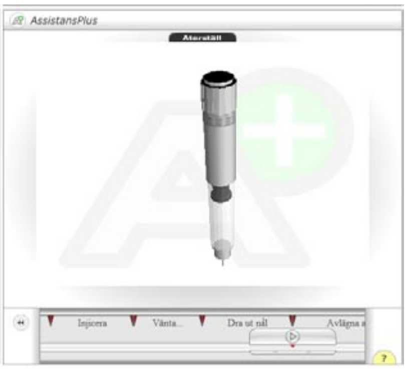
AssistansPlus
Återställ
Injicera
Vänta...
Dra ut nål

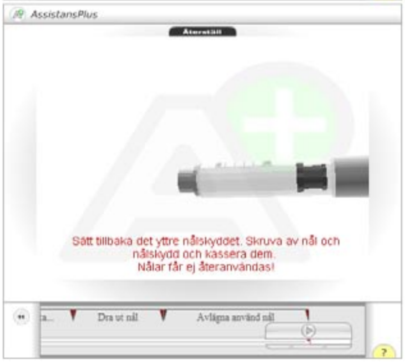
AssistansPlus
Återställ
Sätt tillbaka det yttre nålskyddet. Skruva av nål och nålskydd och kassera dem.
Nålar får ej återanvändas!
Dra ut nål
Avlägna använd nål

Genotropin MiniQuick image sequence:

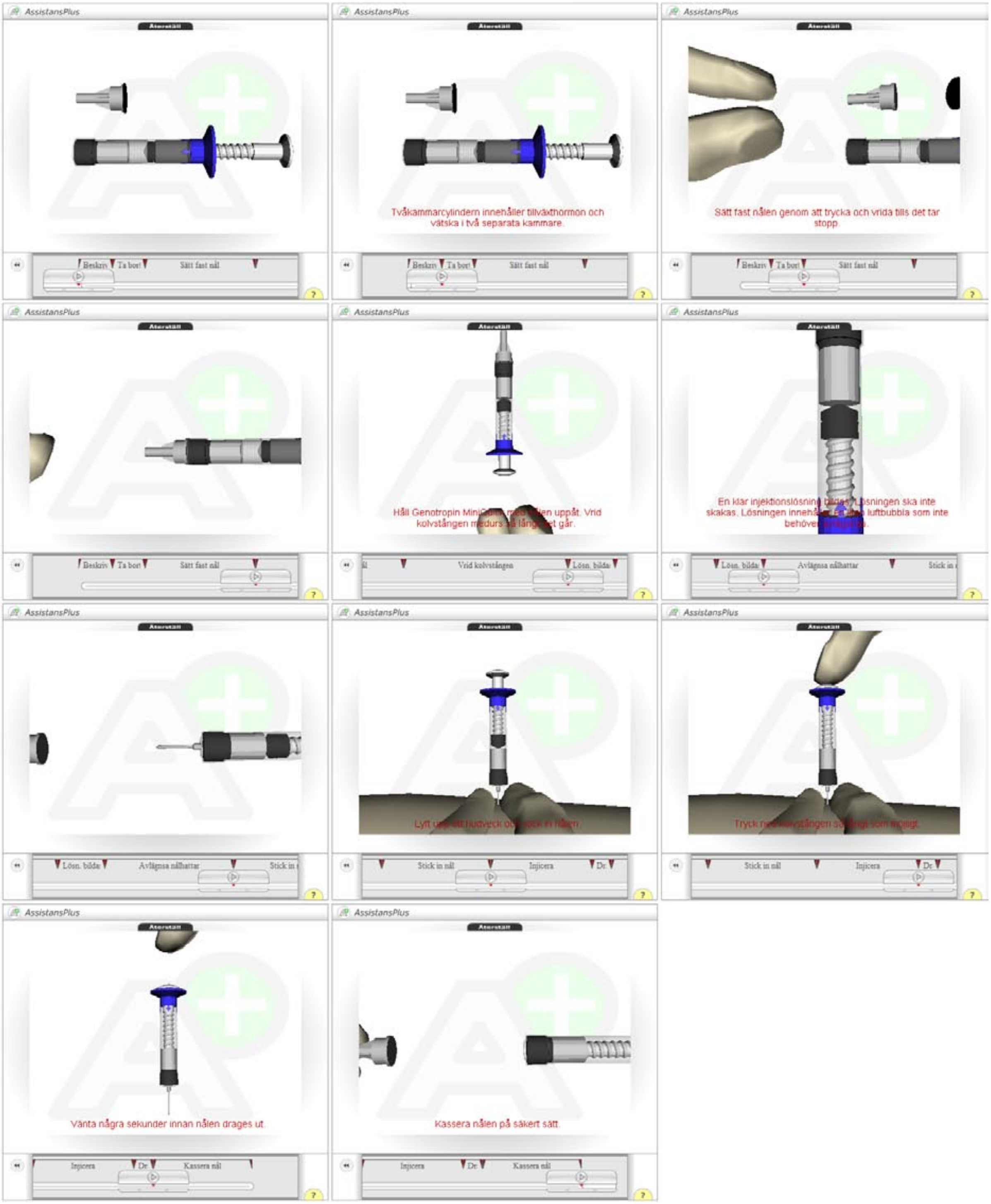

Supplement: Supplementary file 1 [file jmir_v13i3e50_app1.pdf]
