# Supplementary material for: 3D Visualization as a Communicative Aid in Pharmaceutical Advice-Giving over Distance
Source: J Med Internet Res. 2011 Jul 18;13(3):e50. doi: 10.2196/jmir.1437 (PMC3222187; doi:10.2196/jmir.1437)
Supplement: Supplementary file 2 [file jmir_v13i3e50_app2.pdf]

Description of the survey

Thank you for taking the time to participate in this study!

Below are a list of question concerning your experience of the telephone and AssistancePlus advice sessions, respectively. There may be some expressions that are unfamiliar to you in the questions. Here is a short glossary for you to refer to.

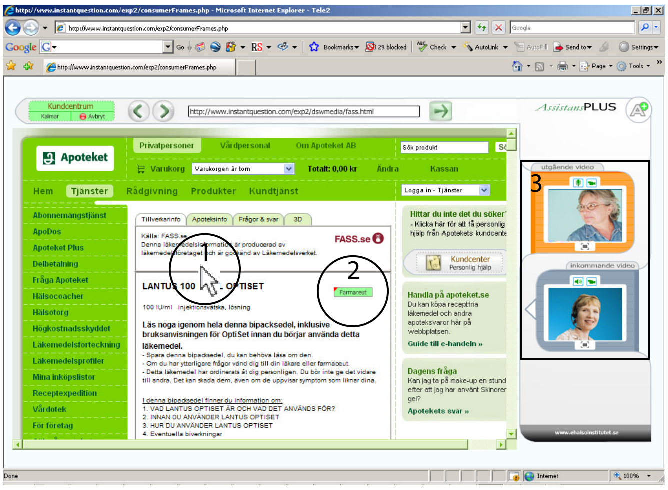

1 - Cursor - the mouse cursor used to point and click on the screen
2 - Remote cursor - displays the position of the pharmacist's cursor.
3 - Video image - Video feed that displays your or the pharmacist's face

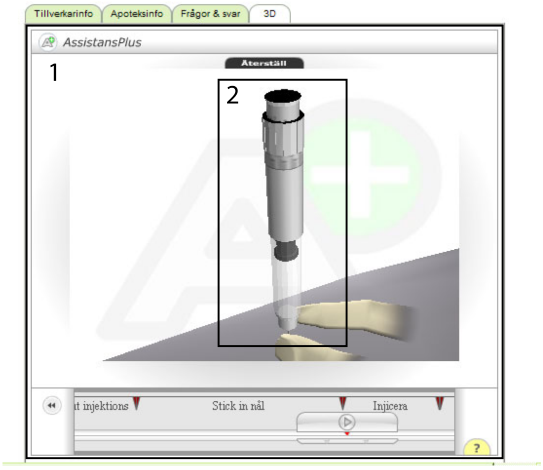

1 - 3D animation - the interactive movie that was used to demostrate the handling instructions for the pharmaceutical product
2 - 3D model - The model representing the pharmaceutical product in the 3D animation

Background information

1 Gender

Man ☐ Woman ☐

2 Age

18-24 ☐ 25-34 ☐ 35-49 ☐ 50-65 ☐ 65+ ☐

3 Would you describe yourself as someone that is eager to try new things?

No, absolutely not ☐ No, not usually ☐ Yes, sometimes ☐ Yes, absolutely ☐ | No opinion ☐

4 Would you describe yourself as having a positive attitude towards computers and the Internet?

No, absolutely not ☐ No, not usually ☐ Yes, sometimes ☐ Yes, absolutely ☐ | No opinion ☐

5 How may times have you used a computer **for work or studies** in the last **month?**

Never ☐ A few times ☐ Several times ☐ Daily ☐ | Do not know/not applicable ☐

6 How many times have you used a computer in **outside work/studies** in the last **month**?

Never	A few times	Several times	Daily	Do not know
☐	☐	☐	☐	☐

7 How many times have you used the **Internet outside work/studies** in the last **month**?

Never	A few times	Several times	Daily	Do not know
☐	☐	☐	☐	☐

Previous experience with pharmaceutical advice services

8 How many times have you in any way sought information about medication in tha last **year**?

Never	1-4 times	5-10 times	More than 10 times	Do not know/prefer not to say
☐	☐	☐	☐	☐

Comparison between telephone and AssistancePlus

9 For each of the following properties, distribute 10 points between telephone and AssistancePlus. A high value indicates a positive rating.The sum of the two values must be 10 (zero values are allowed).

	Property	Telephone		AssistancePlus	
**a**	Easy to use<br>How easy is it to use	☐	+	☐	= 10
**b**	Ease of communication<br>How easy is it to communicate using it	☐	+	☐	= 10
**c**	Sense of personal contact<br>How personal the sense of contact feels	☐	+	☐	= 10
**d**	Explanatory power<br>How difficult/complex issues can it handle	☐	+	☐	= 10
**e**	Understanding<br>How well one understands given information	☐	+	☐	= 10
**f**	Trust<br>How much one trust the given information	☐	+	☐	= 10
**g**	Efficiency<br>How efficient the communication is	☐	+	☐	= 10

AssistancePlus

10 Mark the number that corresponds with how you experienced AssistancePlus.

		1	2	3	4	5	6	7	8	9		Do not know
a	Awful	☐	☐	☐	☐	☐	☐	☐	☐	☐	Fantastic	☐
b	Frustratin	☐	☐	☐	☐	☐	☐	☐	☐	☐	Satisfying	☐
c	Boring	☐	☐	☐	☐	☐	☐	☐	☐	☐	Stimulating	☐
d	Difficult	☐	☐	☐	☐	☐	☐	☐	☐	☐	Easy	☐
e	Powerful	☐	☐	☐	☐	☐	☐	☐	☐	☐	Powerless	☐
f	Inflexible	☐	☐	☐	☐	☐	☐	☐	☐	☐	Flexible	☐
g	Easy to get started	☐	☐	☐	☐	☐	☐	☐	☐	☐	Difficult to get started	☐

Audio/video

11 I find it positive that I could see the pharmacist (the pharmacist's video image)

Disagree completely ☐ ☐ ☐ ☐ ☐ ☐ Agree completely | ☐ Do not know

12 I find it positive that the pharmacist could see me (your own video image)

Disagree completely ☐ ☐ ☐ ☐ ☐ ☐ Agree completely | ☐ Do not know

13 Jag tycker att kvaliteten på videobilderna var bra.

Disagree completely ☐ ☐ ☐ ☐ ☐ ☐ Agree completely | ☐ Do not know

14 Compare the audio quality with AssistancePlus with that on the telephone

	1	2	3	4	5	6	7	8	9		Do not know
Much worse than the telephone	☐	☐	☐	☐	☐	☐	☐	☐	☐	Much better than the telephone	☐

Show and point

15 I valued being able to look at text and pictures on web pages together with the pharmacist.

Disagree completely ☐ ☐ ☐ ☐ ☐ ☐ Agree completely | ☐ Do not know

16 I valued being able to see what the pharmacist was pointing to with his/her remote cursor (the green cursor marked "Pharmacist")

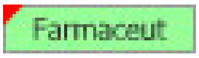

Disagree completely ☐ ☐ ☐ ☐ ☐ ☐ Agree completely | ☐ Do not know

17 Where you aware of that the pharmacist could see your cursor also?

Yes ☐ No ☐ | Did not think about that ☐

18 I valued being able to use my cursor to point to things?

Disagree completely ☐ ☐ ☐ ☐ ☐ ☐ Agree completely | ☐ Do not know

3D animation and the 3D model

The questions in this section pertain only to your experience of the 3D functionality used to demonstrate the pharmaceutical products

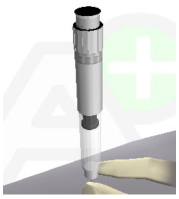

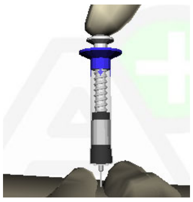

19 I feel that I understood the information that was passed on using the 3D features well.

Disagree completely ☐ ☐ ☐ ☐ ☐ ☐ Agree completely | ☐ Do not know

20 I valued being able to control the 3D content myself.

Disagree completely ☐ ☐ ☐ ☐ ☐ ☐ Agree completely | ☐ Do not know

21 Would you prefer the 3D model that was used to represent the phamarceutical product to be true-to-life or simplified in the way it is presented?

Simplified 1 ☐ 2 ☐ 3 ☐ 4 ☐ 5 ☐ 6 ☐ 7 ☐ 8 ☐ 9 ☐ true-to-life | Vet ej ☐

22 I feel that it would be valuable to be able to use the 3D model on my own after the advice-giving session has been completed.

Disagree completely ☐ ☐ ☐ ☐ ☐ ☐ Agree completely | ☐ Do not know

Feeling of control

23 Did you feel active or passive during the advice-giving session using the telephone and AssistancePlus, respectively?

a - During the telephone session

	1	2	3	4	5	6	7	8	9		Do not know
Passive	☐	☐	☐	☐	☐	☐	☐	☐	☐	Active	☐

b - During the AssistancePlus session

	1	2	3	4	5	6	7	8	9		Do not know
Passive	☐	☐	☐	☐	☐	☐	☐	☐	☐	Active	☐

24 Who did you feel was the one that controlled the dialog during the telephone and AssistancePlus sessions, respectively?

a - During the telephone session

	1	2	3	4	5	6	7	8	9		Do not know
Your yourself	☐	☐	☐	☐	☐	☐	☐	☐	☐	The pharmacist	☐

b - During the AssistancePlus session

	1	2	3	4	5	6	7	8	9		Do not know
Your yourself	☐	☐	☐	☐	☐	☐	☐	☐	☐	The pharmacist	☐

25 I feel satisfied with the way the dialogue was controlled during the telephone session.

Disagree completely ☐ ☐ ☐ ☐ ☐ ☐ Agree completely | ☐ Do not know

26 I feel satisfied with the way the dialogue was controlled during the AssistancePlus session.

Disagree completely ☐ ☐ ☐ ☐ ☐ ☐ Agree completely | ☐ Do not know

Area of usage

27 Choose which channels you would prefer when seeking information about pharmaceutical products for each type of product. High value indicate a positive rating. The sum of the ratings must be 10 (zero values are allowed).

	Telephone		AssistancePlus		Pharmacy store		Internet	
**a** Simple products<br>For advice on simple products, for example non prescription asperin.	☐	+	☐	+	☐	+	☐	= 10
**b** Simple prescription medication<br>For advice on simple types of prescription medications.	☐	+	☐	+	☐	+	☐	= 10
**c** Complex medication<br>For advice on complex medications that do not require any special handling.	☐	+	☐	+	☐	+	☐	= 10
**d** Medication requiring handling<br>For advice on medications or medical aids requiring non-trivial handling.	☐	+	☐	+	☐	+	☐	= 10

Would use

28 Would you use a service such as AssistancePlus where it available on Apoteket's web page?

	1	2	3	4	5	6	7	8	9		Do not know
Not at all likely	☐	☐	☐	☐	☐	☐	☐	☐	☐	Very likely	☐

Open-ended questions

29 Was there anything with AssistancePlus that you found difficult or confusing?

....

....

....

....

30 Do you have any suggestions on how AssistancePlus can be improved?

....

....

....

....

31 What type of questions/issues do you think that AssistancePlus is best suited for?

....

....

....

....

32 Who do you think would use a service like AssistancePlus?

....

....

....

....

33 What would make you NOT WANT to use a service like AssistancePlus?

....

....

....

....

34 Do you have any other comments?

....

....

....

....

Thank you for you contribution!
